# Clinical evaluation of panel testing by next-generation sequencing (NGS) for gene mutations in myeloid neoplasms

**DOI:** 10.1186/s13000-016-0456-8

**Published:** 2016-01-22

**Authors:** Chun Hang Au, Anna Wa, Dona N. Ho, Tsun Leung Chan, Edmond S. K. Ma

**Affiliations:** Division of Molecular Pathology, Department of Pathology, 1/F Li Shu Fan Block, Hong Kong Sanatorium & Hospital 2 Village Road, Happy Valley, Hong Kong, China

**Keywords:** Next-generation sequencing, Acute myeloid leukemia, Gene mutations, *FLT3* internal tandem duplication, Cytogenetics, Bioinformatics

## Abstract

**Background:**

Genomic techniques in recent years have allowed the identification of many mutated genes important in the pathogenesis of acute myeloid leukemia (AML). Together with cytogenetic aberrations, these gene mutations are powerful prognostic markers in AML and can be used to guide patient management, for example selection of optimal post-remission therapy. The mutated genes also hold promise as therapeutic targets themselves. We evaluated the applicability of a gene panel for the detection of AML mutations in a diagnostic molecular pathology laboratory.

**Methods:**

Fifty patient samples comprising 46 AML and 4 other myeloid neoplasms were accrued for the study. They consisted of 19 males and 31 females at a median age of 60 years (range: 18–88 years). A total of 54 genes (full coding exons of 15 genes and exonic hotspots of 39 genes) were targeted by 568 amplicons that ranged from 225 to 275 bp. The combined coverage was 141 kb in sequence length. Amplicon libraries were prepared by TruSight myeloid sequencing panel (Illumina, CA) and paired-end sequencing runs were performed on a MiSeq (Illumina) genome sequencer. Sequences obtained were analyzed by in-house bioinformatics pipeline, namely BWA-MEM, Samtools, GATK, Pindel, Ensembl Variant Effect Predictor and a novel algorithm ITDseek.

**Results:**

The mean count of sequencing reads obtained per sample was 3.81 million and the mean sequencing depth was over 3000X. Seventy-seven mutations in 24 genes were detected in 37 of 50 samples (74 %). On average, 2 mutations (range 1–5) were detected per positive sample. *TP53* gene mutations were found in 3 out of 4 patients with complex and unfavorable cytogenetics. Comparing NGS results with that of conventional molecular testing showed a concordance rate of 95.5 %. After further resolution and application of a novel bioinformatics algorithm ITDseek to aid the detection of *FLT3* internal tandem duplication (ITD), the concordance rate was revised to 98.2 %.

**Conclusions:**

Gene panel testing by NGS approach was applicable for sensitive and accurate detection of actionable AML gene mutations in the clinical laboratory to individualize patient management. A novel algorithm ITDseek was presented that improved the detection of *FLT3*-ITD of varying length, position and at low allelic burden.

## Background

The molecular basis of AML is heterogeneous. Cytogenetic study is well documented as a mandatory test at diagnosis to stratify patients into favorable, intermediate and adverse prognostic categories [1]. The identification of gene mutations in AML allows the further characterization of the molecular heterogeneity of this disease, especially with the subgroup of intermediate risk AML that often exhibit a normal karyotype [2]. In the recommendation by the European LeukemiaNet (ELN), mutations involving three genes *NPM1*, *FLT3* and *CEBPA* are considered in AML prognostication scheme [3]. Cytogenetically normal AML (CN-AML) with mutated *NPM1* without *FLT3*-*ITD*, or mutated *CEBPA*, are incorporated in the favorable genetic group. Recent data shows that only double, but not single, *CEBPA* mutations confer a favorable prognosis [4–7]. Likewise the National Comprehensive Cancer Network (NCCN) Guideline for AML incorporates CN-AML with *NPM1* mutation or isolated *CEBPA* mutation in the absence of *FLT3*-*ITD* in the better-risk status category, whilst CN-AML with *FLT3*-*ITD* mutation is considered poor-risk [8]. The detection of concurrent *KIT* mutation relegates the core binding factor (CBF)-related AML from better-risk to intermediate-risk.

With the sequencing of the AML genome [9], the first cancer genome to be sequenced, mutations involving many more genes important in leukemogenesis are being deciphered. Through the whole genome sequencing approach, the genomic and epigenomic landscapes of adult *de novo* AML [10] and the clonal architecture of secondary AML [11] are comprehensively described. The genetic aberrations can be grouped under nine categories defined according to biological function and a putative role in AML pathogenesis, namely transcription factor fusions, gene encoding nucleophosmin (*NPM1*), tumor suppressor genes, DNA methylation-related genes (*DNMT3A*, *IDH1*, *IDH2* and *TET2*), activated signaling genes, chromatin modifying genes, myeloid transcription factor genes, cohesin complex genes and spliceosome complex genes [10]. The clinical utility of these AML gene mutations is under investigation [12, 13]. Besides serving as powerful prognostic indicators, the mutational profile may have the potential to affect treatment decision in AML. For example, patients in the favorable genetic subgroup of mutated *NPM1* lacking *FLT3*-*ITD* may be considered for post-remission chemotherapy alone without resorting to allogeneic bone marrow transplantation (BMT) [14], and more recently the most favorable treatment response is reported in mutated *NPM1* lacking *FLT3*-*ITD* and harboring *IDH2* R140 mutation [15]. Regarding upfront treatment, *DNMT3A* and *NPM1* mutations together with MLL translocations, predict for an improved outcome with high-dose daunorubicin induction chemotherapy [15]. Moreover, mutations of the DNA methylation genes may predict for response to hypomethylation agents [16], although this issue remains controversial [17]. Finally, these AML gene mutations are themselves novel drug targets, as evident by the clinical trials on the *FLT3* inhibitors [18] and *IDH2* inhibitors [19].

It should be noted that whole genome sequencing and whole exome sequencing are predominantly research tools. To serve clinical management needs, accurate genetic testing results should be reported with a reasonable short turnaround time. We perform clinical evaluation of a gene panel for testing myeloid disorders by NGS in a diagnostic molecular pathology laboratory setting.

## Methods

### Patient samples

Fifty patient samples were accrued from April 2011 to November 2014. They comprised 19 males and 31 females at a median age of 60 years (range 18–88 years). The diagnoses were M0 = 1, M1 = 7, M2 = 12, M3 = 1, M4 = 5, M5 = 3, M6 = 2, not otherwise specified (NOS) = 4, AML with myelodysplasia (MDS) related changes (AML-TMDS) = 5, AML transformed from MDS or MDS/MPD = 3, therapy-related AML = 1, refractory AML = 2, high-grade MDS = 3 and atypical chronic myeloid leukemia = 1. A diagnosis of AML-NOS was rendered in four patients due to diagnosis on peripheral blood only (n = 2), aparticulate bone marrow aspirate (*n* = 1) and diagnosis at another institution (*n* = 1). The study was approved by the Research Ethics Committee of Hong Kong Sanatorium & Hospital (reference number: REC-2015-02).

DNA was extracted from the corresponding specimens (bone marrow = 42 and peripheral blood = 8) using QIAamp DNA Blood Mini Kit (Qiagen, Germany). Four additional samples were available from 3 patients for comparison (buccal swab and second peripheral blood samples of patient 31, post-treatment sample of patient 35 and relapsed sample of patient 36). Extracted DNA was quantified using Qubit dsDNA BR Assay Kit and Qubit 2.0 Fluorometer (Life Technologies, USA).

Any known mutation status of *FLT3*, *NPM1*, *KIT*, *CALR*, *MPL*, *CSF3R* by Sanger sequencing and *JAK2* V617F by allele-specific polymerase chain reaction (PCR) and PCR-restriction fragment length polymorphism (PCR-RFLP) analysis of the patient samples were curated from the clinical record for comparison with NGS results.

### Control samples

Archival DNA samples (*n* = 11) with known mutations in *CALR* (*n* = 6), *JAK2* (*n* = 2), *KIT* (*n* = 1), *MYD88* (*n* = 1) and *TP53* (*n* = 1) were retrieved as positive controls to validate the NGS myeloid panel. DNA extracted from peripheral blood samples of healthy adults with normal complete blood profile (*n* = 18) were accrued in July and October 2014 as negative controls. These control samples were analyzed in the same way as the patient samples.

### NGS myeloid gene panel

The myeloid gene panel targets 54 genes (full coding exons of 15 genes: *BCOR*, *BCORL1*, *CDKN2A*, *CEBPA*, *CUX1*, *DNMT3A*, *ETV6*/*TEL*, *EZH2*, *KDM6A*, *IKZF1*, *PHF6*, *RAD21*, *RUNX1*/*AML1*, *STAG2* and *ZRSR2*, and exonic hotspots of 39 genes: *ABL1*, *ASXL1*, *ATRX*, *BRAF*, *CALR*, *CBL*, *CBLB*, *CBLC*, *CSF3R*, *FBXW7*, *FLT3*, *GATA1*, *GATA2*, *GNAS*, *HRAS*, *IDH1*, *IDH2*, *JAK2*, *JAK3*, *KIT*, *KRAS*, *KMT2A*/*MLL*, *MPL*, *MYD88*, *NOTCH1*, *NPM1*, *NRAS*, *PDGFRA*, *PTEN*, *PTPN11*, *SETBP1*, *SF3B1*, *SMC1A*, *SMC3*, *SRSF2*, *TET2*, *TP53*, *U2AF1* and *WT1*) by 568 amplicons (length range: 225–275 bp). The combined coverage was 141 kb. Amplicon sequencing libraries were prepared from 50 ng of DNA per sample using TruSight myeloid sequencing panel (Illumina, USA). A highly multiplexed pool of oligonucleotide pairs upstream and downstream to each region of interest (ROI) was employed. Each oligonucleotide contained unique target-specific sequences and universal adaptor sequence used in the subsequent amplification reaction. For each sample, an extension-ligation reaction extended across the ROI and followed by ligation to unite the two probes to yield a library of templates with common ends. This library of new templates was PCR amplified with a unique pair of indexes incorporated for downstream sequence-based sample identification. After PCR clean-up, double-stranded DNA length and quantity of individual libraries were assessed by DNA 1000 kit and 2100 Bioanalyzer system (Agilent, USA). Libraries were normalized according to the measured quantity and pooled in batches (8 to 24 libraries per pool). Paired-end sequencing runs were performed on a MiSeq (Illumina, USA) with reagent kit v3 according to manufacturer’s instructions.

### Variant calling and annotation

Paired sequences obtained from each sample were mapped to human genome reference GRCh37/hg19 using BWA-MEM [20] version 0.7.7 with default parameters. Three variant callers were used: (1) Samtools [21] version 0.1.19, with mpileup command parameters -L 100000 -d 100000 to cater for amplicons with depth exceeding 250-fold and bcftools command parameter-m0.99 to use the new insertion-deletion (INDEL) calling model; (2) GATK HaplotypeCaller [22] version 2.8-1 according to the best practices recommended by the authors; and (3) VarScan [23] version 2.3.7 somatic mutation calling mode based on one of the negative controls or the matched germline DNA if available. For detection of *FLT3* internal tandem duplication (ITD), additional variant callers were used specifically for the region chr13:28607161–28609590: (1) Pindel [24] version 0.2.5a7 with the insert size configured as the summation of forward and reverse sequencing read length, to adapt the algorithm to the amplicon sequencing reads in this study, and (2) a novel algorithm ITDseek developed in this study (details described in a separate section).

Variant calls were first annotated by Ensembl Variant Effect [25] Predictor version 75 and then manually examined by at least two individuals. Sequence alignment of selected variants was manually examined with Integrative Genomics Viewer [26] (IGV). ROI sequencing depth was summarized using BEDTools [27] version 2.19.1 Minimum reportable variant allele frequency (AF) is 10 % of sequencing depth at least 500-fold. ROI with depth less than 500-fold were regarded as sub-optimal regions. Variants found to be reported in COSMIC database version 67 and/or dbSNP version 138 were prioritized for manual examination while those reported in 1000 Genomes project (phase 1) were excluded. *FLT3* ITD and *ASXL1* c.1934dupG mutations were confirmed in selected patients using PCR fragment analysis by capillary electrophoresis (primer sequences available upon request) and analysis software Peak Scanner version 1.0 (http://www.appliedbiosystems.com). Variants were described according to the recommendations of Human Genome Variation Society (HGVS). Variant descriptions were checked by Mutalyzer Name Checker (http://mutalyzer.nl).

### Evaluation of *FLT3* ITD detection algorithms and development of a novel detection algorithm ITDseek

*FLT3 ITD* detection performance of Pindel, Samtools, GATK HaplotypeCaller were compared based on a simulated dataset of ITD mutations. An *in silico FLT3* ITD sequencing read simulator ITDsim was developed based on BioPerl [28]. The amplicon targeting chr13:28,608,112–28,608,312 (both primer binding sites excluded) was chosen as the simulation target due to its highest reported *FLT3* mutation rate in COSMIC among the three amplicons in the region. All combinations (*n* = 40,401) of ITD lengths (range: 1–201 bp; *n* = 201) and starting positions (chr13:28,608,112–28,608,312; *n* = 201) were simulated separately. ITD allelic burden was defined as 50 %. For each combination, 1000 paired-end ITD reads and 1000 paired-end wild-type reads (both 2 × 275 bp) were simulated and the FASTQ file pair was subject to variant calling as described above. Simulation and corresponding variant calling were performed on a Cray XC30 supercomputer.

To overcome the difficulty in calling long ITD mutations with short-read NGS amplicon sequencing, a novel *FLT3* ITD detection algorithm ITDseek was developed based on the following principles. It takes SAM/BAM alignments as input and outputs any detected ITD mutations in VCF format. In case of a short ITD (e.g., 9 bp) present in the middle of raw sequencing reads (e.g., 250 bp), the wild-type sequences upstream and downstream of ITD mutation are long enough for proper alignment to the *FLT3* locus, with an insertion in between representing the additional sequence (operation “I” in CIGAR field of SAM alignment output by BWA-MEM). General-purpose variant callers like Samtools, GATK HaplotypeCaller and VarScan could then readily identify the inserted nucleotides. In contrast, if a given ITD is too long and/or too close to either end of amplicon, the sequence downstream of ITD mutation will become too short or even completely absent in the raw sequences obtained. Without long enough downstream sequences, the additional duplicated nucleotides are marked as soft-clipped instead (operation “S” in CIGAR field). These soft-clipped nucleotides are usually ignored by general-purpose variant callers as if they are sequencing adapter. In principle, realignment of soft-clipped nucleotides is a way to identify possible ITD mutations. Since BWA-MEM outputs a separate secondary alignment representing those soft-clipped nucleotides, such realignment is effectively performed. However, secondary alignments are usually ignored by general-purpose variant callers. ITDseek specifically searches for any soft-clipping points in primary alignments and correlate them with any corresponding secondary alignments for ITD identification. The length of ITD was extrapolated by the distance between the point of soft-clipping and the beginning of realignment of soft-clipped bases. The analysis is performed in individual reads and directions separately to identify any rare ITD clone (as rare as a single read only) or multiple ITD clones. In case of a special *FLT3* ITD type that the additional sequence is entirely insertion of unknown origin, there is no secondary alignment. ITDseek identifies such ITD by comparing points of soft-clipping in sequencing read pairs. ITDseek was also evaluated based on the same simulated dataset described before. Source codes and documentation of ITDsim and ITDseek are available at http://github.com/tommyau/ITDseek for non-commercial use.

## Results

### High concordance of NGS results versus known mutations status

The NGS panel showed 100 % concordance in detecting all 5 single-nucleotide mutations and 6 INDEL mutations from 11 corresponding positive control samples. The single-nucleotide mutations were *JAK2* c.1849G > T p.Val617Phe (*n* = 2), *KIT* c.2447A > T p.Asp816Val, *MYD88* c.794 T > C p.Leu265Pro and *TP53* c.916C > T p.Arg306* (all *n* = 1). The INDEL mutations included insertions of 5 bp (*CALR* c.1153_1154insCTTGT, c.1154_1155insTTGTC) and deletions of 19 bp (*CALR* c.1124_1142del), 34 bp (*CALR* c.1103_1136del) and 52 bp (*CALR* c.1099_1150del; *n* = 2). No mutation was detected from all 18 negative control samples.

Next, we compared NGS results with known mutation status of 42 patient samples from conventional testing (*n* = 111 results, both positive and negative), including *FLT3* ITD (*n* = 39), *FLT3* tyrosine kinase domain (TKD; *n* = 39), *NPM1* (*n* = 22), *JAK2* (*n* = 4), *KIT* (*n* = 4), *CALR*, *MPL* and *CSF3R* (all *n* = 1). The concordance rate was 95.5 % (106 of 111), being observed in 17 mutation positive results (*NPM1 n* = 8, *FLT3* TKD *n* = 5, *FLT3* ITD *n* = 3, *KIT n* = 1) and 89 mutation negative results (*FLT3* TKD *n* = 34, *FLT3* ITD *n* = 32, *NPM1 n* = 14, *JAK2* and *KIT* both *n* = 3, *CALR*, *CSF3R* and *MPL* all *n* = 1). The 5 discrepancies were *JAK2* V617F (*n* = 1) and *FLT3* ITD (*n* = 4) mutations missed by NGS as elaborated below.

**Fig. 1:**
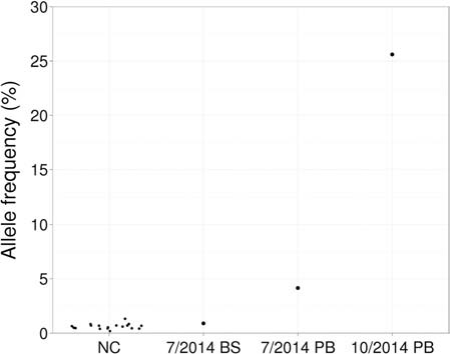
*JAK2* V617F allele frequencies as measured by next-generation sequencing. Samples included 18 negative controls (NC) and 3 samples of patient 31, namely buccal swab (BS) and peripheral blood (PB) samples in July 2014, and peripheral blood sample in October 2014.

For patient 31, both allele-specific PCR and PCR-RFLP analysis of the initial sample (July 2014) showed *JAK2* V617F weakly positive result (Fig. 1). No such mutation was reported by the NGS panel on the same sample. We asked whether the mutation was actually detected by NGS but not reported based on the reportable minimum variant AF (10 %). To obtain the AF of *JAK2* V617 regardless of variant callers, we checked aligned reads at the corresponding genomic position chr9:5073770G > T. V617F AF was 4.1 % in the initial sample, 0.9 % in matched buccal swab sample and 25.6 % in the second peripheral blood sample (October 2014) (Fig. 1). The mean noise level was 0.6 % based on 18 negative controls (0.2–1.3 %, 99 % confidence interval 0.45–0.79 %; Fig. 1). The slight but significant elevation of *JAK2* V617F AF in the initial sample showed the mutation was indeed detectable by NGS and confirmed the weakly positive results by conventional molecular analysis.

Known *FLT3* ITD mutations detected by Sanger sequencing in patients 3, 36, 37 and 41 were originally not reported by the NGS panel. However, lengths of the ITD mutations (30–189 bp, *n* = 4) fell within the range reported to be detectable by Pindel (17–185 bp) [29], which was one of the variant callers used in this study. In the study by Spencer et al. employed sonication-based DNA fragmentation during sequencing library preparation, start positions of aligned reads were randomly distributed [29]. In contrast, start positions of aligned reads in this study were fixed by primers of corresponding PCR amplicons. We hypothesized that the ITD detection sensitivity also depended on the relative positions of ITD within corresponding amplicons.

### Comprehensive bioinformatics evaluation of *FLT3* ITD detection methods

We evaluated the performance of variant callers for ITD detection when the mutation position varied along the amplicon. A simulation approach was chosen to encompass a wide range of possible ITD mutations regardless of actual sample availability. A read simulator ITDsim was developed to simulate 2 × 275 bp sequencing data for ITD mutations at various starting positions, based on the amplicon with highest reported *FLT3* ITD mutation rate (chr13:28,608,112–28,608,312) in the region. Given that the length of amplified region was 201 bp, starting positions included all these 201 positions and mutation length ranged from 1 to 201 bp (total 40401 combinations). For each combination of starting position and length, a pair of FASTQ files was simulated with sequencing depth 2000X and ITD AF 50 %. To determine the best possible performance of ITD detection, the simulated *FLT3* sequencing reads were perfect-matching sequences derived from the GRCh37/hg19 reference genome without errors and all other amplicons were not simulated. Bioinformatics analysis of the simulated data was performed in the same way as the clinical samples.

**Fig. 2:**
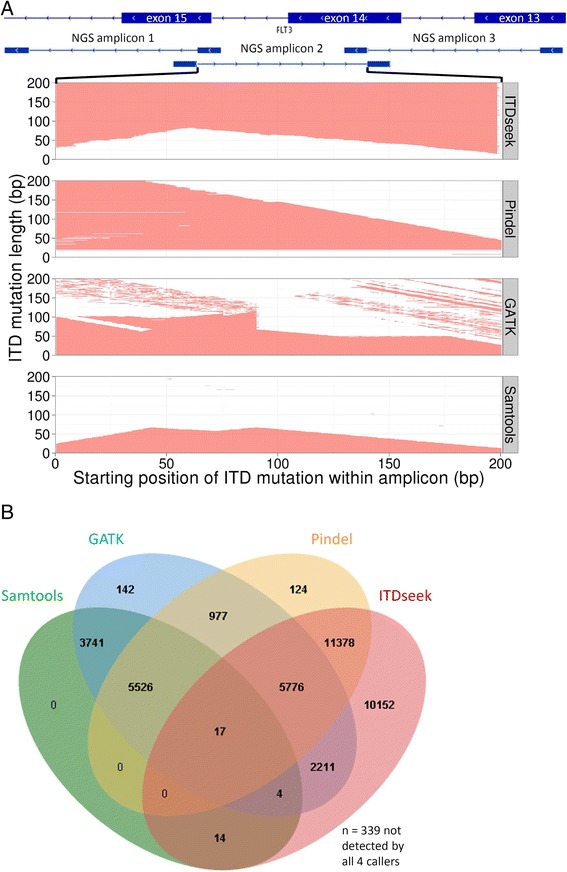
*FLT3* ITD detection performance of a novel algorithm ITDseek versus Pindel, GATK HaplotypeCaller and Samtools. **a** Detected combination of ITD length and relative position in the *FLT3* NGS amplicon 2 (chr13:28,608,112-28,608,312) was indicated by red shading in the corresponding panel of each caller. **b** Venn diagram showing the number of ITD alleles detected by any or none of the four tested callers.

Pindel, GATK HaplotypeCaller and Samtools detected 58.9, 45.5 and 23.0 % of simulated ITD mutations, respectively (Fig. 2a). Although Pindel detected most ITD mutations among the three callers, the maximum length of detected ITD mutation decreased from 201 bp to 45 bp at the 41^st^ nucleotide and 201^st^ nucleotide of the amplicon, respectively (Fig. 2a). ITD detection sensitivity was demonstrated to depend on not only length but also its relative position in the amplicon.

### A novel *FLT3* ITD detection algorithm ITDseek

We therefore closely examined the BWA-MEM alignments and observed two major differences between wild-type and ITD sequencing reads: (1) longer sequences became soft-clipped (ignored for analysis including variant calling), compared with consistently short soft-clipped sequences representing universal adapters, and (2) there was additional alignment of part of the soft-clipped sequences to the same *FLT3* locus, but marked as supplementary alignment that was ignored for analysis in a similar manner as soft-clipping. Based on these observations, a novel ITD mutation algorithm ITDseek was developed and detected 73.1 % of the simulated ITD mutations (Fig. 2a). For most starting positions in the amplicon (1 to 198), although the minimum length of detected ITD mutation ranged from 15 to 83 bp, the maximum detection length was consistently 201 bp (the longest simulated length). ITDseek was insensitive to the remaining 3 starting positions (199–201 bp).

More importantly, ITDseek detected 96.8 % (10152 of 10491) of combined false negatives by the other three callers (Fig. 2b). ITDseek increased overall detection rate from 74.0 % (other three callers) to 99.2 % (all four callers). Computation requirement of ITDseek was minimal that it took <20 s and <2GB RAM to analyze a full MiSeq V3 run of 8 samples by 8 CPU cores (5-year-old Intel Xeon 2.8GHz processors).

**Fig. 3:**
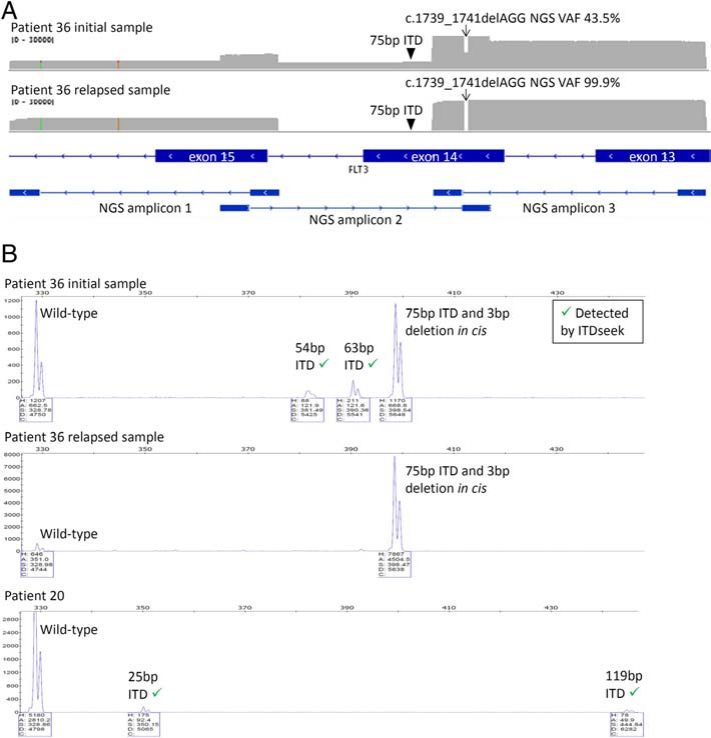
*FLT3* of patients 36 and 20 as characterized by next-generation sequencing and fragment analysis. **a** NGS sequencing depth histogram for 3 amplicons covering *FLT3* exons 13 to 15 at scale 0–30000X. Magnitude of drops in sequencing depth at amplicon 2 and the region of 3 bp deletion (c.1739_1741delAGG; indicated by arrow) was proportional to the deletion VAF as indicated. The amplicon 2 covered the 75 bp ITD (indicated by triangle) but was affected by the 3 bp deletion *in cis*. **b** PCR fragment analysis for *FLT3* ITD detection. ITD of 54 bp, 63 bp (patient 36 initial sample), 25 bp and 119 bp (patient 20) detected by NGS and ITDseek were confirmed by the corresponding fragments. Single additional fragment detected in both initial and relapsed samples of patient 36 confirmed the additional allele, which consisted of 75 bp ITD and 3 bp deletion *in cis* and was not detected by NGS due to allele drop-out.

After including ITDseek as an additional variant caller, known *FLT3* ITD mutations of patients 3 (189 bp), 37 (30 bp) and 41 (30 bp) were detected to resolve 3 discrepancies. Regarding the remaining patient 36, ITDseek detected two different ITD mutations of lower AF (63 and 54 bp) but not the known 75 bp ITD mutation. A 3 bp deletion c.1739_1741delAGG located near the extension/ligation oligo binding site of amplicon 2 was also detected by the NGS panel (Fig. 3a). Reanalysis of original Sanger sequences confirmed the 3 bp deletion and suggested that it was *in cis* with 75 bp ITD mutation. Sanger sequencing of relapsed sample showed that the two *in cis* mutations became almost the only *FLT3* allele detected. NGS of that relapsed sample detected the 3 bp deletion at VAF 99.9 %. NGS sequencing depth of amplicon 2 dropped from 5500X (initial sample) to 70X (relapsed sample), which was consistent with complete drop-out of the ITD allele (Fig. 3a). PCR fragment analysis of the original and relapsed samples supported these observations (Fig. 3b).

In patient 20, which was reported ITD negative by Sanger sequencing, ITDseek detected two ITD mutations (119 and 25 bp). PCR fragment analysis confirmed both mutations (Fig. 3b). The allelic burden (1.4 and 3.2 %, respectively) was below the detection limit of Sanger sequencing. Taken together, after further resolution and application of ITDseek, there were still 2 samples (patients 20 and 36) with discrepancies and concordance was 98.2 % (109 of 111). *FLT3* mutation detection was more sensitive by NGS since ITDseek detected 2 additional ITD alleles with low VAF in both patients 20 and 36, notwithstanding the one complex allele in patient 36 that was undetected by NGS due to allele drop-out.

### NGS mutation profile

**Fig. 4:**
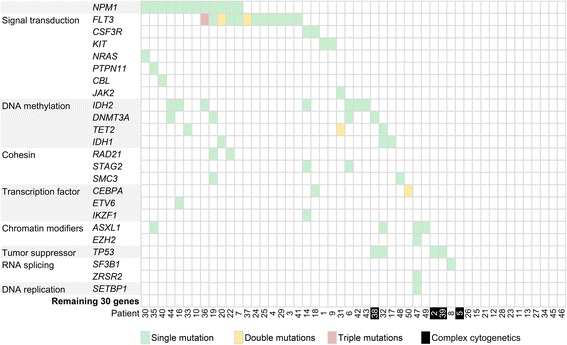
Mutation status matrix across 50 patient samples and 54 genes. Patients (initial samples only, if applicable) and genes are arranged in columns and rows, respectively. Seventy-seven detectable mutations in 24 genes are represented by colored boxes (*green*, *yellow* and *red* for 1, 2, and 3 mutations, respectively). Patient samples with known complex and unfavorable cytogenetics are shaded in black.

The mean count of sequencing reads obtained per sample was 3.81 million (range 2.02–8.51 million) and the mean sequencing depth was over 3000X. Seventy-seven mutations in 24 genes (Table 1) were detected in 37 of 50 patient samples (74 %). On average 2 mutations (range 1–5) were detected per positive sample. Mutations were detected in the following genes: *FLT3* (*n* = 16), *NPM1* (*n* = 12), *IDH2* (*n* = 7), *ASXL1*, *DNMT3A*, *TET2*, *TP53* (all *n* = 4), *CEBPA*, *IDH1* (both *n* = 3), *CSF3R*, *KIT*, *RAD21*, *SMC3*, *STAG2* (all *n* = 2), *CBL*, *ETV6*, *EZH2*, *IKZF1*, *JAK2*, *NRAS*, *PTPN11*, *SETBP1*, *SF3B1* and *ZRSR2* (all *n* = 1). The two most frequently mutated genes in our patient cohort, *FLT3* and *NPM1*, were in keeping with the mutational frequency of genes in AML as reported in the literature (Fig. 4) [30]. Based on mechanism of action, genes involved in signal transduction (*FLT3*, *CSF3R*, *KIT*, *NRAS*, *PTPN11*, *CBL* and *JAK2*) and DNA methylation (*DNMT3A*, *TET2*, *IDH1* and *IDH2*) were the most frequent mutated groups, accounting for 24 (31 %) and 18 (23 %) out of 77 detectable mutations [31].

**Table 1:** Summary of 50 patients in this study.

No.	Sex/Age	Diagnosis	Cytogenetics	Conventional molecular testing	NGS myeloid panel	Clinical outcome
1	M/56	AML-NOS^*^	Not done	FLT3-	KIT p.D816V	Alive on treatment
2	F/37	M1	Near-tetraploid and complex	NPM-, FLT3-	TP53 p.E62^*^	Treated in Mainland China
3	M/60	High grade MDS	Normal	FLT3-ITD (189 bp), del(5q) FISH-	FLT3 ITD (189 bp)	Alive in CR after BMT
4	F/83	M6	No growth	FLT3-ITD-TKD+	FLT3 p.D835Y	Died
5	M/58	High grade MDS	Complex > 3 abnormality	NPM1-, FLT3-	Negative	Died after BMT
6	M/57	AML-TMDS	Normal	NPM1-, FLT3-	DNMT3A p.E423^*^	Died of relapse after BMT
					IDH2 p.R140Q	
					STAG2 p.R259^*^	
7	F/66	AML-TMDS	Normal	NPM1+, FLT3-ITD-TKD+	FLT3 p.D835Y	Died of CNS disease
					NPM1 p.W288Cfs*12	
8	F/49	Refractory AML	Not done	FLT3-	SF3B1 p.K666E	Died
9	F/35	M2	inv(16)	KIT+	KIT p.D816V	Alive in CR after BMT
10	F/44	M4	Normal	NPM+, FLT3-	NPM1 p.W288Cfs*12	Alive in CR
11	F/82	M5a	No growth	FLT3-	Negative	Died
12	F/25	M2	No growth	AML1-ETO-, NPM1-, FLT3-, KIT-	Negative	Alive in CR
13	F/76	AML transformed from MDS	No growth	FLT3-, del(5q) FISH-, BCR-ABL1-	Negative	Died
14	F/52	M1	+14	NPM1-, FLT3-	CSF3R p.T816I	Lost to follow up
					IKZF1 p.R111^*^	
					IDH2 p.R140Q	
					STAG2 p.R1012^*^	
15	M/82	M1	add(3q), add(7q), +8,	FLT3-, BCR-ABL-	Negative	Died
16	F/59	M6	Poor growth	NPM1+, FLT3-	NPM1 p.W288Cfs*12	Died
					ETV6 p.Y346^*^	
					IDH2 p.R140W	
17	F/78	M2	+10	NPM1-, FLT3-, AML1-ETO-, PML-RARA-	IDH1 p.R132C	In CR with vidaza and died of second cancer
18	M/61	AML transformed from CMML	Poor growth	FLT3-, del(5q) FISH-, PDGFRB FISH-, BCR-ABL-	CSF3R p.T618I	Died
					CEBPA p.Y67^*^	
19	F/64	AML-NOS^b^	Normal	NPM1+, FLT3 ITD-TKD+	NPM1 p.W288Cfs*12	Died
					FLT3 p.D835Y	
					DNMT3A p.W313^*^	
					SMC3 c.3105 + 2 T > C	
					RAD21 p.Q132^*^	
20	M/54	M1	Normal	NPM1+, FLT3-	NPM1 p.W288Cfs*12	Alive in CR
					IDH1 p.R132H	
					FLT3 ITD (25 bp)	
					FLT3 ITD (119 bp)	
21	M/44	M1	Constitutional inv(9)	FLT3-, BCR-ABL-	Negative	Died. History of HD treated by BEACOPP
22	F/44	M2	Not done	FLT3 ITD + TKD-	NPM1 p.W288Cfs*12	Died
					RAD21 c.1161 + 2 T > A	
					FLT3 ITD (72 bp)	
23	F/33	M2 therapy-related	Poor growth	NPM1-, FLT3-	Negative	Died. History of treated breast cancer
24	F/26	M3	No growth	PML-RARA(s)+, FLT3 ITD + TKD-	FLT3 ITD (33 bp)	Alive in CR
25	M/65	M5a	+8	NPM1-, FLT3 ITD + TKD-	FLT3 ITD (54 bp)	Died
26	F/74	M0	+22	CBFB-MYH11- NPM1- FLT3- BCR-ABL1-	Negative	Alive in CR and maintained on monthly vidaza
27	F/77	AML-TMDS	+8	Not done	Negative	Lost to follow up. History of lung cancer
28	M/18	M2	t(8;21),-Y	AML1-ETO+, KIT-, NPM1-, FLT3-	Negative	Alive in CR
29	F/61	AML-NOS^a^	Poor growth	NPM1-, FLT3 ITD-TKD+, BCR-ABL-, AML1-ETO-	FLT3 p.D835Y	Alive in CR
30	F/39	M2	+8	AML1-ETO-, NPM+, FLT3-	NPM1 p.W288Cfs*12	Alive in CR
					NRAS p.G12D	
31	M/70	M1	Normal	NPM-, FLT3-, PDGFRB FISH-, FIP1L1-PDGFRA-, BCR-ABL-,	TET2 p.S1848^*^	NR to vidaza. Further treatment in Mainland China
					TET2 p.G1152E	
				JAK2 V617F weak+	JAK2 p.V617F (VAF 4.1 %, below original reportable threshold)	
32	F/80	M2	del(5q), +8	Not done	IDH1 p.R132C	Alive and on vidaza
					TET2 p.K693Nfs*18	
					TP53 p.R249S	
					ASXL1 p.W960^*^	
33	F/68	M4	Normal	FLT3-	NPM1 p.W288Cfs*12	Difficult CR and on vidaza
					TET2 p.L346Rfs*2	
34	F/24	M2	t(8;21)	AML1-ETO+, BCR-ABL-, PML-RARA-, NPM1-, FLT3-	Negative	Alive in CR
35	M/60	M5a	+8	Not done	NPM1 p.W288Cfs*12	Alive on treatment
					PTPN11 p.G503A	
					ASXL1 p.G646Wfs*12	
36	F/62	M1	Normal	AML1-ETO-, BCR-ABL1-, PML-RARA-, NPM1+, FLT3 ITD+ *in cis* with 3 bp deletion (c.1739_1741delAGG)	NPM1 p.W288Cfs*12	CR for 1 year. Relapsed on treatment
					FLT3 p.Q580_V581delinsL (c.1739_1741delAGG)	
					FLT3 ITD (54 bp)	
					FLT3 ITD (63 bp)	
					IDH2 p.R140Q	
37	F/46	M4	Near-tetraploid	AML-ETO-, BCR-ABL1-,	FLT3 p.D835Y	Responded to sorafenib and HHT. Received BMT from sibling donor and on treatment
				FLT3 ITD+ (30 bp), FLT3 TKD+	FLT3 ITD (30 bp)	
38	M/66	AML-TMDS	Complex > 3 abnormalities	FLT3-, BCR-ABL1-, JAK2 V617F-	DNMT3A p.M801Nfs*11	Alive on vidaza. History of lung cancer
					TP53 p.R175H	
39	F/62	AML-M5 post-BMT relapse	Complex > 3 abnormalities	Not done	TP53 p.Y220C	Died
40	M/62	M5a transformed from MDS	Normal	NPM1+, FLT3-, MLL FISH-	NPM1 p.W288Cfs*12	Died
					CBL c.1096-1G > T	
41	F/78	AML-TMDS	add(21q)	FLT3 ITD + TKD-	FLT3 ITD (30 bp)	Died
42	M/88	AML-NOS^*^	Not done	Not done	IDH2 p.R140Q	Alive on palliative care
43	M/63	M2	Normal	Not done	IDH2 p.R172K	Alive on vidaza
44	F/60	M2 and bone marrow fibrosis	Normal	5/7/del(20q) FISH-, BCR-ABL-, FLT3-, JAK2 V617F/CALR/MPL-	DNMT3A c.855 + 1G > T	Alive on treatment
					NPM1 p.W288Cfs*12	
					IDH2 p.R140Q	
45	F/28	M4Eo	inv(16)	CBFB FISH+, CBFB-MYH11 PCR+	Negative	Alive in CR
46	F/84	M4Eo	Not done	CBFB-MYH11 PCR+, FLT3-, NPM1-, KIT-	Negative	Died
47	M/51	Atypical CML	Normal	CSF3R-, BCR-ABL-, JAK2 V617F-, PDGFRB FISH-, PDGFRA FISH-	EZH2 c.1852-2A > G	Died
					SETBP1 p.D868N	
					ASXL1 p.G646Wfs*12	
					ZRSR2 p.Q103*	
48	F/53	M2	t(8;21)	KIT-	SMC3 p.S674_R675insL	Alive in CR
49	M/65	High-grade MDS	Normal	Not done	ASXL1 p.G646Wfs*12	Alive on vidaza + eltrombopag trial
50	M/51	M2	Normal	NPM1-, FLT3-, AML1-ETO-	CEBPA p.R343Afs*79	Alive on treatment
					CEBPA p.K313dup	

*CR* complete remission, *NR* non-remission, *FISH* fluorescence in-situ hybridization, *vidaza* 5-azacytadine, *HHT* homoharringtonine, *HD* Hodgkin lymphoma, *PML*-*RARA*(*s*) short isoform of fusion transcript from PML bcr3 breakpoint, *VAF* variant allele frequency, *FLT3* testing included detection of both ITD and TKD

^*^Diagnosis on PB only

^a^Aparticulate aspirate and diagnosis by immunophenotyping only

^b^Diagnosis in another hospital

The small patient number in our cohort precluded meaningful correlation between gene mutations with clinical features and outcome or survival analysis. This notwithstanding, *TP53* mutations were found in three out of four patients (75 %) with complex and unfavorable cytogenetics. The mutational spectrum of the cytogenetic unfavorable group was rather different from that of the cytogenetic normal or intermediate group in our patient cohort. Interestingly, both *ASXL1* p.G646Wfs*12 and *NPM1* p.W288Cfs*12 mutations were detected in the initial sample of patient 35 but not detected in the post-treatment sample. Although whether the two mutations occurred in the same or independent clones is unknown, they represented one of the first documented exceptions to mutual exclusion nature of *ASXL1* and *NPM1* mutations in AML [32].

## Discussion

While infrequent in CN-AML, mutations affected *TP53* is associated with a complex karyotype [33], as confirmed by our patient cohort. This may define a distinct subgroup of AML that displays primary resistance to therapy and a very dismal prognosis [34, 35]. It is unclear whether *TP53* mutations cause and promote increasing cytogenetic instability, or whether these are secondary mutations occurring after the onset of chromosomal instability. One study showed that a subset of patients with complex karyotype did not have *TP53* mutations, whilst all *TP53*-mutated AML were found in complex karyotype, suggesting that complex karyotype preceded *TP53* mutations [36]. Also most mutations of *TP53* were associated with del(17p) [36], supporting the contention that *TP53* mutations were the second hit in leukemogenesis. More recently, *TP53* mutations associated with therapy-related AML were shown to be present before the exposure to chemotherapy, suggesting that a pre-leukemic clone harboring TP53 mutation gained survival advantage after chemotherapy, rather than induced by chemotherapy [37]. Hence TP53-mutated AML and therapy-related AML may have more in common than previously recognized.

Detection of *FLT3* ITD is an important test for CN-AML due to its impact on prognosis and treatment [38]. Highly variable length, allelic burden and number of the ITD mutations were observed [39]. These characteristics pose a challenge in detection by next-generation sequencing, specifically not the sequencing process per se, but the bioinformatic analysis of the short sequences obtained (<300 bp). Pindel was shown to be the state-of-the-art ITD variant caller, particularly its detection of ITD alleles with length up to 185 bp [29]. However, our study showed that Pindel detected 3 ITD alleles of length 33 bp (patient 24) to 72 bp (patient 22) but not 4 ITD alleles of length 30 to 189 bp. Similarly, a recent study showed that Pindel detected 14 ITD alleles of length up to approximately 60 bp but not 3 ITD alleles of length approximately 50, 90 and 110 bp [40].

By comprehensive evaluation based on 40401 simulated ITD alleles with length up to 201 bp, we demonstrated that detection from amplicon-based NGS data was dependent on relative ITD position within amplicon, in addition to ITD length. Other laboratories may also use the simulator ITDsim to evaluate the performance of their own bioinformatics pipeline. We also developed a novel detection algorithm ITDseek because recently developed tools including BreaKmer [41] and Genomon ITDetector [42] were developed for sequencing library prepared with random DNA fragmentation and similar to Pindel. Amplicon Indel Hunter [43] was developed for amplicon-based sequencing data but the actual implementation was not available for parallel evaluation. ITDseek detected most false negatives (97 % of simulated samples) of Pindel, GATK and Samtools. For the actual samples in this study, ITDseek detected ITD alleles up to 189 bp missed by Pindel. ITDseek was designed to process the de facto standard BAM alignment file with minimal computation time (<20 s for a whole MiSeq run by 8 CPU cores) and is expected to be easily incorporated in various bioinformatics pipelines of other laboratories.

## Conclusions

We show that gene panel testing by NGS approach in a diagnostic molecular pathology laboratory allows sensitive and accurate detection of actionable AML gene mutations to individualize patient management. The diagnostic approach to AML is facing a paradigm shift in the genomics era [44]. As more targeted therapy become available, the greater is the clinical demand for comprehensive molecular profiling. The results of genomic study hold promise for better understanding disease pathogenesis and classification, refining prognostic stratification and uncovering new drug targets. Further studies should focus on the clinical utility of the genomics to document whether this approach translated into improvements in AML patient outcome and survival [45]. The novel algorithm ITDseek presented in this paper improves the detection of *FLT3*-ITD in the laboratory setting of amplicon-based next-generation sequencing.
